# Relapsing Fever

**Published:** 1852-08

**Authors:** 


### ART. IV. — *Relapsing Fever.* By THE EDITOR.

In the distribution of cases for analytical investigation hitherto, I have practically assumed that they were either of the *Typhus* or *Typhoid* type. Are all the varieties or species of Continued Fever embraced in these two forms of the disease? This is an interesting and important question which perhaps cannot, in the present state of knowledge, be answered positively.

The distinctive nosological features of *Typhus* and *Typhoid* fever have been but recently established, and it would not be surprising if further investigation should lead to other subdivisions of continued fever, showing that we have been accustomed to include in one or both of these forms, affections which should be considered distinct.

The opinion has been advanced by some British writers that already there are sufficient grounds for such a subdivision. A form of fever observed at different epochs in Edinburg and London is described as possessing, in common, characters differing from those of *Typhus* and *Typhoid fever* sufficiently to constitute it a distinct affection, which has been named, *Relapsing fever.* The following extract from an able article in the *British and Foreign Medico-Chirurgical Review*, No. for July, 1851, furnishes a brief historical notice of the occurrence at different times and places of the kind of fever which has received this title:

* "In the early part of 1843, a febrile disease appeared in Edinburgh and Leith, so different in its course, in its symptoms, and in the amount of its mortality, from any Continued Fever which had been recently observed there, that it was at once and unhesitatingly declared to be a peculiar and new disease. It was soon known that the same disease had appeared in Glasgow a month or two prior to its outbreak in Edinburgh; it was more or less prevalent also in Dundee and in other large towns in Scotland, whether it appeared in London or in other English towns is doubtful; at any rate it was not described. It was observed with great accuracy, and recorded in the periodicals of the time, by Craigie, Alison, Arnott, Henderson, Halliday, Douglas, Jackson, and Mackenzie; and it was made the subject of two special and excellent treatises by Cormack and Wardell.

"It was soon discovered, however, that although this fever had not been seen in Edinburgh for many years, it was not altogether a new disease. Dr. Christison expressed an opinion that it was similar to the fever witnessed by

---

* We omit the various authorities to which the writer refers. For these the reader may consult the Review.

him in 1817 – 18, and recorded by Welsh, in his well-known work, and by himself in the 'Library of Practical Medicine.' That this opinion is correct, and that the Edinburgh epidemic of 1817 – 18, was in great measure made up of this disease, no one can doubt, who will attentively collate the descriptions of Welsh and Christison, with those furnished by the observers of the attack in 1843. But not only in Edinburgh was evidence of its former existence brought forward. It was observed that it had evidently formed part of the Irish fever of 1817 – 18 – 19, which had been so minutely ecorded by Barker and Cheyne. It appeared also that this fever had been prevalent in Ireland for many years. Epidemics in 1739 and 1741 were described in unequivocal terms by Rutty; and other epidemics during the eighteenth century, and those in Dublin in 1806 and 1826, presented, among other forms of fever, the peculiar and unmistakable features of the disease in question.

"The fever thus distinguished and elaborately described by the Scotch observers in 1843, became again epidemic in Glasgow, Edinburgh, and other towns in Scotland, in 1847. It did not by any means prevail so extensively, and there was a simultaneous occurrence of other species of fever; among which, however, the eyes of Steele, Paterson, Orr, and others, trained by the epidemic of 1843, had no difficulty in singling out this peculiar form. In 1847 it was also epidemic in London; and having come under the observation of Dr. Jenner, has been very carefully described. Before this time, in 1846, and in previous years, sporadic cases had been witnessed in the metropolis; and since 1847 a case has every now and then presented itself at the London Fever Hospital, and at other institutions.

"Instructed by the experience of these observers, it is also easy to perceive that this disease is not confined to these islands. It appears, although imperfectly described and confounded with other forms, in the pages of the celebrated treatise of Hildenbrand; and in the epidemic, in 1847, in Upper Silesia, which we intend presently to describe, it evidently formed in some places the great bulk of the cases. Yet it must be said, that although its characters are so striking that the most superficial observer could not overlook them, the German systematic writers have made no allusion to it as a separate disease; and even those who observed it have failed to draw that obvious inference to which the Scotch physicians unanimously came, viz., that it is a disease altogether distinct from ordinary Continued Fever.

"We are not aware that any perfectly satisfactory evidence is to be found in French writers of the existence of this fever in France; it has, in fact, considering the elaborate descriptions of the Scotch physicians, been somewhat singularly overlooked in that country, as in Germany. In America it is not, I believe, known, or at any rate it has not been described. There is reason from Dr. Bell to believe, that it is known in Persia."

The subject of *Relapsing fever* has, as yet, received little or no attention in this country. At the time of writing my second report, I confess I was only aware of the fact that this name had been employed to designate a form of continued fever. I was not acquainted with the distinctive characters attributed to it.

In reference to that Report the reader will perceive that among the forty-eight cases analyzed, were several (fifteen) which were characterized by the occurrence of a *relapse of fever*, after convalescence appeared to be distinctly declared.* The occurrence of this second febrile career in so large a proportion of that collection of cases, was the more surprising from the fact that nothing of the kind had been observed in the cases previously analyzed, and I can now add that the same is true, as a general remark, of the cases upon the analysis of which my Third Clinical Report is based. The article in the British and Foreign Medico-Chirurgical Review, from which the above extract was taken, appeared about the time I had completed my second report. After reading that article the inquiry naturally arose whether the cases of fever characterized by the occurrence of relapses in the collection I had just analyzed, might not be cases of *Relapsing fever*. With reference to this inquiry I now propose, as I then promised, to subject them to a separate analysis, with a view to ascertain if, in other points than the occurrence of relapses, they differ from *Typhoid* and *Typhus* fever, and, if, at the same time, they exhibit traits which are said to distinguish *Relapsing fever*. Before entering on this analysis, however, inasmuch as it will probably not be an act of injustice to our readers to presume that many, if not most of them are but little acquainted with the subject, I will premise some account of the diagnostic points which are said to distinguish this form of fever. In so doing I shall avail myself, without giving more particular credit, of the article in the British and Foreign Medico-Chirurgical Review already referred to, and a paper by Dr. William Jenner, on the "identity or non-identity of the specific cause of Typhoid, Typhus and Relapsing fever," contained in the Medico-Chirurgical Transactions, published by the Royal Medical and Chirurgical Society of London." [Second series vol. 15, 1850.]

Evidences of a form of fever supposed to be identical with *Relapsing fever*, have heretofore received different names, such as the "short fever;" the "five day fever;" the "seven day fever;" "bilious remittent fever;" "remittent icteric fever;" "mild yellow fever," etc.

The disease is stated to affect all ages, and both sexes in about an equal ratio.

The *access* does not present any very distinctive features. The attack is oftener abrupt than in Typhoid fever, and the muscular and articular pains are apt to be severe.

Delirium, and other head symptoms are represented to be oftener absent, and, when present, less in degree than in the other forms of continued fever.

The absence of the abdominal symptoms which are so generally present, in a greater or less degree, in *Typhoid* fever, is an important point distinguishing *Relapsing fever.* Diarrhœa, with notable meteorism, and tenderness in the iliac regions, do not belong to the latter. It is, however, characterized by certain symptoms pertaining to the digestive system rarely found in *Typhoid*, viz: nausea and vomiting, which are frequently prominent symptoms, and tenderness over the epigastrium. The matter vomited is stated to be "bright grass green," and sometimes like coffee grounds, approximating to the black vomit of yellow fever.

The eruptions characteristic of *Typhus* and *Typhoid*, are not found in *Relapsing* fever. Sudamina and occasionally petechiæ have been observed. An eruption consisting of "small spots, round, purple, unaltered by pressure" has been described by some observers as an element of the affection. These spots are stated to be extremely like flea bites, and it does not appear to be fully ascertained that they were not, in fact, flea bites. The chest symptoms, i. e. cough and bronchial rales, so generally present in *Typhoid* fever, are said to be less commonly present in this disease. *Epistaxis*, so frequently observed in *Typhoid* fever, sometimes occurs. The pulse, seldom falling below 100, in more than half the cases is 120, and in a considerable number the frequency is still greater.

Profuse sweating is stated to occur pretty uniformly, preceding the apparent convalescence, and also when the relapse of fever is about to terminate. Another distinguishing feature referable to the skin, is more or less yellowness, occurring on the fourth or fifth day. In severe cases the jaundice is often a prominent symptom. The most distinctive event, however, is that indicated by the title of the disease, to wit: the occurrence of *relapses.* The first febrile career continues for a period rarely less than *four*, nor more than *ten* days, and then ends, generally after profuse sweating, which is considered to be critical, leaving the patient free from febrile movement, and apparently convalescent. After an interval varying in duration from *five* to *eight* days, another attack occurs, generally abruptly, and frequently preceded by a chill. The recurring febrile movement is equally or more severe than the first. It continues from four to five days, and then terminates usually after profuse sweating. Usually after a single relapse, the patient becomes permanently convalescent. A second, third, and even a greater number of relapses, however, have been observed to occur.

The disease is seldom fatal. Patients almost uniformly recover, unless some complication takes place, such as pleurisy, pneumonitis or dysentery.

The most distinctive characters derived from autopsical examinations, are the absence of the intestinal lesions belonging to *Typhoid* fever. The peyerian glands are unaffected. The spleen is generally enlarged and softened.

It may be communicated by contagion, and Dr. Jenner, in the article already referred to, has collected observations tending to show that Relapsing fever cannot be derived from patients laboring under the other forms of Continued Fever, but that it alone produces the special miasm for its propagation. How far the facts contributed by him go to establish this important point, it does not fall within my present purpose to inquire.

This form of fever does not secure immunity from the other forms; nor from subsequent attacks of the same form.

These are, briefly, the more prominent of the traits distinctive of *Relapsing* fever which are mentioned by the two writers to whom I am indebted for the foregoing summary. They appear to have been deduced from an examination of reports of epidemics, supposed to be of this character, by various authors. If they are sufficient to render probable the position that the disease to which they relate is a peculiar form of fever, distinct from the *Typhus* and *Typhoid* forms, it is certainly desirable that collections of cases should be subjected to careful numerical analyses. The question can only be definitively settled by the results of such analyses.

So far as I know, we have no account of a form of fever prevailing in this country, analogous to the relapsing fever as just described. Were the cases characterized by relapse, among those observed by me during the winter of 1850–51, cases of *Relapsing* fever? With a view to arrive at the answer to this question, and, if the conclusion be in the affirmative, to furnish a small contribution toward the natural history of that form of fever, I will now proceed to analyze the cases referred to.

*Analysis of Fifteen Cases of Continued Fever, characterized by Relapses.*

In my Second Clinical Report I have stated that *fifteen* of the *forty-eight* cases upon the analysis of which that Report was based, were characterized by the occurrence of relapses. Of these fifteen cases *nine* were included among those of *Typhoid* fever; *one* was considered a case of *Typhus*, and *five* were among the cases of *Doubtful type.* Upon a fresh examination of the cases I find that one case in the *Typhoid* group was rejected from the list in consequence of the first convalescence occurring about the time the patient was admitted into the hospital. I am satisfied that this case may be included in the category. The single case of *Typhus* characterized by a relapse, was undoubtedly a case of that type, the characteristic eruption being well marked. The apparent convalescence in this case occurred on the *eleventh* day, and the relapse of fever was of two days duration, after an interval of only three days. These facts show that this could not have been a case of *Relapsing* fever, and I shall accordingly now exclude it. The whole number of cases, therefore, will remain the same as stated in the Second Report, viz: fifteen; and of these fifteen cases *ten* were distributed among those of *Typhoid* fever, and the remaining *five* among the cases of *Doubtful type.*

Of the fifteen cases, *eleven* were males, and *four* were females.

The age of the youngest patient was *nine* years; of the oldest, *thirty-five* years. The average age was a fraction over *twenty* years.

All the patients were from Ireland. *Six* had recently arrived in this country—i. e. within the space of five weeks. *Six* had lived in this country several months—i. e. between six and sixteen months. *One* had resided in this country *four*, and *one* five years. In one case the period of residence is not stated.

*Two* of the cases were admitted in the month of October; *four* during the month of November; *eight* in December; *one* in January, and none in February and March, my term of service ending with the latter month.

*Access.* The duration of the access was not ascertained in *seven* cases. Of the remaining *eight* cases, the attack was sudden in *four;* there was an access of a single day in *one* case, and of *two* days in *two* cases. The duration of the access here, as heretofore, is measured by the period elapsing from the commencement of illness, to the time of taking to bed.

Of *thirteen* cases in the histories of which the presence or absence of a chill, or of chills is noted, the access was ushered in by one or more in *twelve.* The chill was followed by perspiration in *seven,* and was not thus followed in *three* cases.

Pain in the head, back and limbs was complained of during the access, or at the onset of the disease in *nine* cases; in the head alone in *three* cases; in the head and limbs in *one* case, and in the back only in *one* case.

Of thirteen cases in which the presence or absence of vomiting during the access, or at the commencement, was noted, it existed in *eight*, and was absent in *five*.

Diarrhœa is not noted to have been present during the access, or at the commencement, in a single instance.

*Three* of the patients stated that they had previously had an attack of Continued Fever.

*Two* of the patients were sons of the patient affected with *Typhus* fever, in which an imperfect relapse occurred, this case being that already referred to which was included among the cases characterized by relapse in the Second Report.

*General Aspect.* Passive capillary congestion of the face was noted in all cases but one, and in the excepted instance the congestion was active, in other words the face was flushed. The passive congestion was considerable in *three* cases, moderate in *eight* cases, and slight in *three* cases.

The eyes were more or less injected in *five* cases; in *one* case there was no injection; and with respect to the remaining cases nothing is stated relative to this point.

Yellowness of the conjunctiva existed in two cases. These were the only two cases in which this symptom was noted out of the forty-eight cases composing the collection upon the analysis of which the Second Report was based.

*Nervous System.* Of *fourteen* cases there were no manifestations of delirium in *five*, and more or less delirium existed in *nine*. The manifestations of delirium consisted in incoherency only, in one case. In the remaining eight cases there were efforts to get out of bed. In several instances, however, the delirium was of very brief duration. In three cases it was manifested only on a single night. In not a single case was this sympton at all prominent.

Deafness is noted but in *two* cases.

*Digestive System.* In *ten* cases there was nothing like diarrhœa. Of the remaining *five* cases the evidences of diarrhœa consisted in *one* case of too frequent dejections for the first three days, the patient having taken a dose of salts the night before entering the hospital; in one case the dejections were too frequent on the fifth, sixth and seventh days; in two cases this was the fact on a single day only. In but one case was diarrhœa a symptom of any prominence. These results are remarkable when it is considered that *ten* of the cases were embraced in the *Typhoid*, and none in the *Typhus* group. More or less *meteorism* existed in *nine*, and this symptom was absent in *six* cases. The meteorism was moderate in *four*, and slight in *four* cases. In *one* case only was it considerable.

More or less *abdominal tenderness* was present in all but three cases.

There was tenderness in the right iliac region in *ten* cases. In all of these ten cases the degree of tenderness was slight, and in several instances it was present only for a short period, in some but for a single day. *Tenderness over the epigastrium* existed in *six* cases. By reference to the Second Clinical Report it will be seen that of the forty-eight cases upon the analysis of which that Report was based, epigastric tenderness was noted in six instances. Thus all the cases in which it was present in that collection were characterized by relapses! The tenderness was universal over the abdomen in *one* case.

Vomiting is noted to have occurred in but three cases ; this is exclusive of the access or commencement of the disease, to which reference, as to this symptom, has been already made.

*Eruption.* Each of the fifteen cases was unattended by an eruption. This result is certainly remarkable. Recollecting that ten of the cases were included in the cases of *Typhoid* fever upon the analysis of which my Second Clinical Report was based, it is interesting to refer to the facts contained in the section of that Report devoted to eruptions. It is there stated as follows: "The characteristic eruption was present in *twelve* of the *twenty-nine Typhoid* cases. This result differs from that obtained by the first analysis. The latter developed a larger proportion of cases in which this symptom was present, viz.: in *twenty-three*, of *thirty* cases; the ratio, thus, being *two-thirds*, while in the present collection it is less than *one-half.* I can offer no explanation of the above disparity."

If we were to deduct from the twenty-nine Typhoid cases in the second collection, the ten cases characterized by relapses, in which there was no eruption, the ratio of the cases in which the eruption was present would be nearly the same as in the first collection — the disparity with respect to this symptom would disappear; or, in another point of view, of the seventeen cases in the second collection in which no eruption existed, *ten* may be suspected to have been cases of Relapsing fever. The disparity in comparing the second with the third analysis, as respects the eruption, will be found to be even more striking.

*Respiratory Apparatus.* Epistaxis was noted in *six* cases. Pneumonitis existed in *three* cases. In two cases, spasmodic inspiration occurred. Aside from these facts, there was nothing worthy of note referable to the respiratory system.

*Circulation.* The average mean frequency of the pulse was a fraction over 75. The highest mean in any case was 108; the lowest, 69.

As respects the average mean frequency, it was considerably less in these cases than in the *Typhoid* cases in either collection; and the disparity is still greater when compared with the cases of *Typhus*.

*Skin.* Sweating occurred in ten of twelve cases, the histories of which contain information on this point. The sweating was profuse in *eight* cases. It was noted but once in nine cases, and three times in the remaining case.

In each of the cases in which sweating occurred, it took place at a period more or less remote from the date of the first or second convalescence.

Moisture of the surface, i. e., a degree of perspiration less than sweating, was noticed in twelve of fourteen cases. It occurred on one day in *six* cases, twice in *two* cases, and on several days in *four* cases.

Both sweating and moisture, thus, were present in a large proportion of cases; but, it is a point to be noticed, that they did not occur as critical events.

*Duration.* The average duration of the febrile career, from the time of taking to the bed to the date of the first apparent convalescence, was about *nine* days. The longest period was *twelve* days, and the shortest *six* days.

The average duration of the interval between the date of the first apparent convalescence, and the recurrence of fever, was precisely five days. The longest interval was *eight* days; the shortest *three* days.

The average duration of the second febrile career was *six* days. In one case, it is noted that the relapse of fever lasted only two days. Exclusive of this case, the shortest period was *four* days, and the longest *ten* days.

*Mortality.* All the cases terminated in recovery.

*Sequelæ.* Nothing coming under this head worthy of note is contained in the histories.

### *Concluding Remarks.*

On reviewing the results developed by the foregoing analysis, and comparing them with the distinctive traits of *Relapsing fever*, as presented in the account preliminary to the analysis, the correspondence is certainly striking. The access was abrupt in one-half the cases in which this point was ascertained, and unusually short in duration in the remainder. In 5–14 of the cases, there were no evidences of delirium; and in all the cases, this, together with other head symptoms, was slight.

In 10–15 of the cases there was entire absence of diarrhœa, and in one case only was this symptom in any degree prominent or persistent. Vomiting was not a prominent feature, being present at the access or commencement in but 8–13, and afterward in but three instances. Iliac tenderness was very slight in every case, and in six cases tenderness over the epigastrium, which is extremely rare in *Typhoid* and *Typhus* fever, was marked. Meteorism was considerable in but a single instance, and absent in several cases. The abdominal symptoms, thus, were absent or slight in a remarkable degree, unless the cases were of the *Typhus* type, and it is to be borne in mind that none of the cases were considered to be of that type. The chest symptoms, and the circulation offer nothing worthy of particular note, except that the frequency of the pulse was considerably less than is stated to belong to the history of Relapsing fever. Sweating and moisture occurred in a very large proportion of the cases, in this respect presenting a contrast with the average occurrence of these symptoms in *Typhus* and *Typhoid* fever; but differing from other observations with respect to *Relapsing* fever in the fact that these symptoms did not occur at the time of the temporary or permanent convalescence. Mild jaundice, a very rare event in *Typhus* or *Typhoid* fever, was present in *two* of the fifteen cases. But the most striking of all the points of correspondence relate, first, to eruptions, and, second, to the relapses. In none of the cases was there an eruption, a fact which would certainly be very remarkable with respect to the same number of cases of the *Typhus* or *Typhoid* type occurring successively. The circumstances pertaining to the relapses, viz., the duration of the first febrile career, of the interval, and of the second febrile attack, accord with the previous description of these events as they are stated to occur in *Relapsing fever.* Finally, the absence of a fatal tendency is exemplified by the fact that all the cases ended in recovery.

In view of these facts the conclusion seems unavoidable that the cases of fever characterized by relapses, among those which came under my observation in 1850—51, presented the distinctive traits attributed to *Relapsing fever* sufficiently marked to entitle them to be ranked in the class of cases which have been described by different observers as a peculiar form of Continued Fever. This conclusion does not necessarily involve the position that the traits distinguishing the cases authorize their separation as cases of an affection entirely distinct from *Typhus* or *Typhoid* fever. It of course follows either that this position is correct, or that the *Typhoid* or *Typhus* forms of Continued Fever occasionally exhibit, as peculiar modifications, the symptoms which are considered the diagnostic features of *Relapsing fever* by those who regard the latter as a separate form of the disease. I am not prepared to discuss the relative merits of these inferences.

